# An analysis of medication incident reports in the elderly population in Beaumont Hospital

**DOI:** 10.1186/1753-6561-9-S1-A1

**Published:** 2015-01-14

**Authors:** HL Lis, D Williams

**Affiliations:** 1 Royal College of Surgeons in Ireland, 123 St. Stephen’s Green, Dublin 2. Ireland; 2 Department of Geriatrics and Stroke Medicine, Beaumont Hospital, Dublin 9. Ireland

## Background

Medication errors are the single most preventable cause of patient harm that occurs in a medical facility. From prescription to administration, there is a vast amount of opportunity for errors to occur. This emphasizes the need to evaluate the extent of medication error in the elderly as medication errors are estimated to be the fifth most common cause of death among hospitalised elderly population. This study analysed medication incidents reported in the Elderly population in Beaumont hospital and also the trends in reporting, drug classes reported with high frequency, as well as the severity/significance of the incidents reported.

## Methods

The Beaumont Hospital Medication Incident Report Database was the source of data utilised for this study [1]. The inclusion criteria are patients over 65 years old. IT access and software packages were used to analyse the trend in reporting, the drug classes reported, and the severity/significance of the incidents reported (as qualified by an objective rating scale, NCC MERP). A comparison to the criteria outlined in BEERS criteria was performed.

## Results

A total of 157 medication incidents were reported. Incidents were discovered and reported mainly by nurses and pharmacist, at 57% and 34.4% respectively. Only 6% of incidents were reported by doctors. The highest number of incidents occurred in the administration stage, at 49% of cases reported. Incidents at the prescribing stage accounted for 39.5% of incidents. The remaining stages included monitoring, ordering, dispensing, and storage of medication accounted for 11.5% in total. With regards to the medications involved, they were categorised based on the BNF. The highest number of incidents was the cardiovascular system, with 39 incidents. This was followed by infectious disease, at 27 incidents, and the central nervous system, at 26 incidences. Other systems had relatively lower number of incidents. In terms of outcome of incidents, only a small number of patients came to harm, whereas majority either did not reach patients or was reversible. Regarding to Beer’s Criteria, 2 of the drugs used that recommended direct avoidance, and 4 drugs were used that recommended avoidance in certain circumstances.

## Conclusions

In conclusion, the overall pattern of reporting, process stage, and drug classes involved is in line with international findings. Patients were minimally harmed by the incidents. Beer’s criteria were adhered to in general, unless special consideration required. This study reflects the reporting culture in an Irish hospital and allows issues to be addressed to decrease the rate of medication incidents.
